# IgA EGFR antibodies mediate tumour killing *in vivo*

**DOI:** 10.1002/emmm.201201929

**Published:** 2013-08-05

**Authors:** Peter Boross, Stefan Lohse, Maaike Nederend, Johannes Hendrik Marco Jansen, Geert van Tetering, Michael Dechant, Matthias Peipp, Louise Royle, Li Phing Liew, Louis Boon, Nico van Rooijen, Wim K Bleeker, Paul W H I Parren, Jan G J van de Winkel, Thomas Valerius, Jeanette H W Leusen

**Affiliations:** 1 Immunotherapy Laboratory, Laboratory for Translational Immunology, UMC UtrechtUtrecht, The Netherlands; 2 Division of Stem Cell Transplantation and ImmunotherapyDepartment of Internal Medicine II, Christian-Albrechts-UniversityKiel, Germany; 3 Department of Internal Medicine IVNephrology and Hypertension, Christian-Albrechts-UniversityKiel, Germany; 4 Ludger LtdOxfordshire, United Kingdom; 5 BiocerosUtrecht, The Netherlands; 6 Department of Molecular Cell Biology, Vrije University Medical CenterAmsterdam, The Netherlands; 7 GenmabUtrecht, The Netherlands

**Keywords:** antibody therapy, EGFR, Fcalpha receptor I, IgA, tumour immunology

## Abstract

Currently all approved anti-cancer therapeutic monoclonal antibodies (mAbs) are of the IgG isotype, which rely on Fcgamma receptors (FcγRs) to recruit cellular effector functions. *In vitro* studies showed that targeting of FcαRI (CD89) by bispecific antibodies (bsAbs) or recombinant IgA resulted in more effective elimination of tumour cells by myeloid effector cells than targeting of FcγR. Here we studied the *in vivo* anti-tumour activity of IgA EGFR antibodies generated using the variable sequences of the chimeric EGFR antibody cetuximab. Using FcαRI transgenic mice, we demonstrated significant *in vivo* anti-tumour activity of IgA2 EGFR against A431 cells in peritoneal and lung xenograft models, as well as against B16F10-EGFR cells in a lung metastasis model in immunocompetent mice. IgA2 EGFR was more effective than cetuximab in a short-term syngeneic peritoneal model using EGFR-transfected Ba/F3 target cells. The *in vivo* cytotoxic activity of IgA2 EGFR was mediated by macrophages and was significantly decreased in the absence of FcαRI. These results support the potential of targeting FcαRI for effective antibody therapy of cancer.

The study reveals that IgA antibodies directed against EGFR and engaging Fcalpha receptor (FcαRI) on effector cells, have *in vivo* anti-cancer activity. These data support the development of novel immunotherapeutic strategies based on targeting FcαRI.

## INTRODUCTION

Therapeutic monoclonal antibodies (mAbs) are successfully used in the clinic to treat various malignancies. Cetuximab and panitumumab are antibodies that target the epidermal growth factor receptor (EGFR) and are currently part of standard regimens against metastatic colorectal cancer with wild-type (WT) K-Ras. Cetuximab is also approved by the FDA against head and neck cancer (Kim, 2009). Cetuximab has a dual mode of action: both Fab- and Fc-mediated anti-tumour mechanisms were described (Bleeker et al, 2004; Peipp et al, 2008a). The direct Fab-mediated effects involve blocking of ligand binding (Li et al, 2005), prevention of receptor dimerization, which is essential for EGFR-mediated signal transduction (Li et al, 2005) and receptor modulation (Sunada et al, 1986). Indirectly, the Fc part of EGFR antibodies is able to recruit immune-mediated effector functions, such as antibody-dependent cell-mediated cytotoxicity (ADCC) through binding to Fc receptors or complement-dependent lysis (CDC; Peipp et al, 2008a). The importance of ADCC is supported by association of polymorphisms in FcγRs with clinical responses to antibody treatment (Bibeau et al, 2009). The role of FcγR-mediated effector functions during EGFR therapy was also shown in a preclinical model (Overdijk et al, 2011). CDC requires the presence of more than one EGFR antibody recognizing different epitopes and is therefore unlikely to contribute to the *in vivo* mechanism of action of individual EGFR antibodies (Dechant et al, 2008).

Currently, all antibodies approved for human treatment are of the IgG isotype, owing to their long half-life in serum and established manufacturing processes. EGFR antibodies of the IgG1 of IgG2 subclass bind efficiently to activating FcγRs, such as FcγRIIIa or FcγRIIa, resulting in potent ADCC induction. IgG antibodies, however, may co-engage the inhibitory FcγRIIb on several effector cell types, which can downregulate effector functions (Clynes et al, 2000; Hamaguchi et al, 2006; Minard-Colin et al, 2008). In addition, on polymorphonuclear granulocytes (PMNs) binding of IgG1 to the signalling-incapable FcγRIIIb can decrease its *in vivo* activity (Peipp et al, 2008b). Therefore, an alternative antibody format that exploits the maximal killing potential of blood-resident effector cells may improve treatment efficacy.

IgA is best known for its anti-microbial function and is abundantly present at mucosal sites as dimeric or secretory IgA. Monomeric IgA1 is the second most prevalent antibody class in the circulation (Bakema & van Egmond, 2011). Through binding to FcαRI (CD89), IgA can exert potent pro-inflammatory effector functions, such as induction of oxidative burst, phagocytosis and ADCC (Monteiro & van de Winkel, 2003). Tumour cell killing by bispecific antibodies (bsAbs) engaging both the tumour antigen and FcRs was more efficient when FcαRI was targeted over FcγRI (Dechant et al, 2002; Elsasser et al, 1999; Stockmeyer et al, 2000). This is in line with the finding that triggering FcαRI on PMNs results in stronger effector functions than triggering FcγRI, most likely due to more efficient pairing with the FcRγ-chain in the transmembrane domain (Otten et al, 2007). Recently, IgA variants of the chimeric IgG1 EGFR antibody cetuximab were generated and were shown to mediate efficient tumour lysis *in vitro* using human effector cells (Dechant et al, 2007; Lohse et al, 2012). When whole blood was used in the killing assay, IgA2 EGFR induced better tumour cell killing than cetuximab (Dechant et al, 2007). This is most likely because IgA2 EGFR efficiently recruits PMNs, the most abundant effector cell population in the blood that express FcαRI (Monteiro & van de Winkel, 2003). These results suggest that IgA represent an attractive isotype for immunotherapy.

The anti-tumour activity of IgA EGFR antibodies has not been tested *in vivo* before. This is partly due to difficulties in the production and purification of IgA antibodies. In addition, mice do not express FcαRI, and therefore effector functions cannot be accurately studied in WT mice. Here, we have used human FcαRI transgenic (Tg) mice that express FcαRI in a physiological distribution (van Egmond et al, 2000). We demonstrate potent anti-tumour activity of IgA2 EGFR *in vivo* using A431 tumour cells in both a lung and peritoneal xenograft model in severe combined immune deficiency (SCID) mice. IgA2 EGFR also mediated efficient anti-tumour activity in a lung metastasis model using B16F10-EGFR cells in immunocompetent mice. In addition, in a short syngeneic peritoneal model, using EGFR-transfected Ba/F3 cells, IgA2 EGFR induced stronger cytotoxicity than cetuximab. Depletion of different effector populations revealed that the *in vivo* IgA2 EGFR activity was mediated by macrophages. Tumour cell killing was abolished or significantly decreased in the absence of FcαRI.

## RESULTS

### IgA EGFR antibodies mediate tumour cell killing by mouse effector cells *ex vivo*

**Figure 1:**
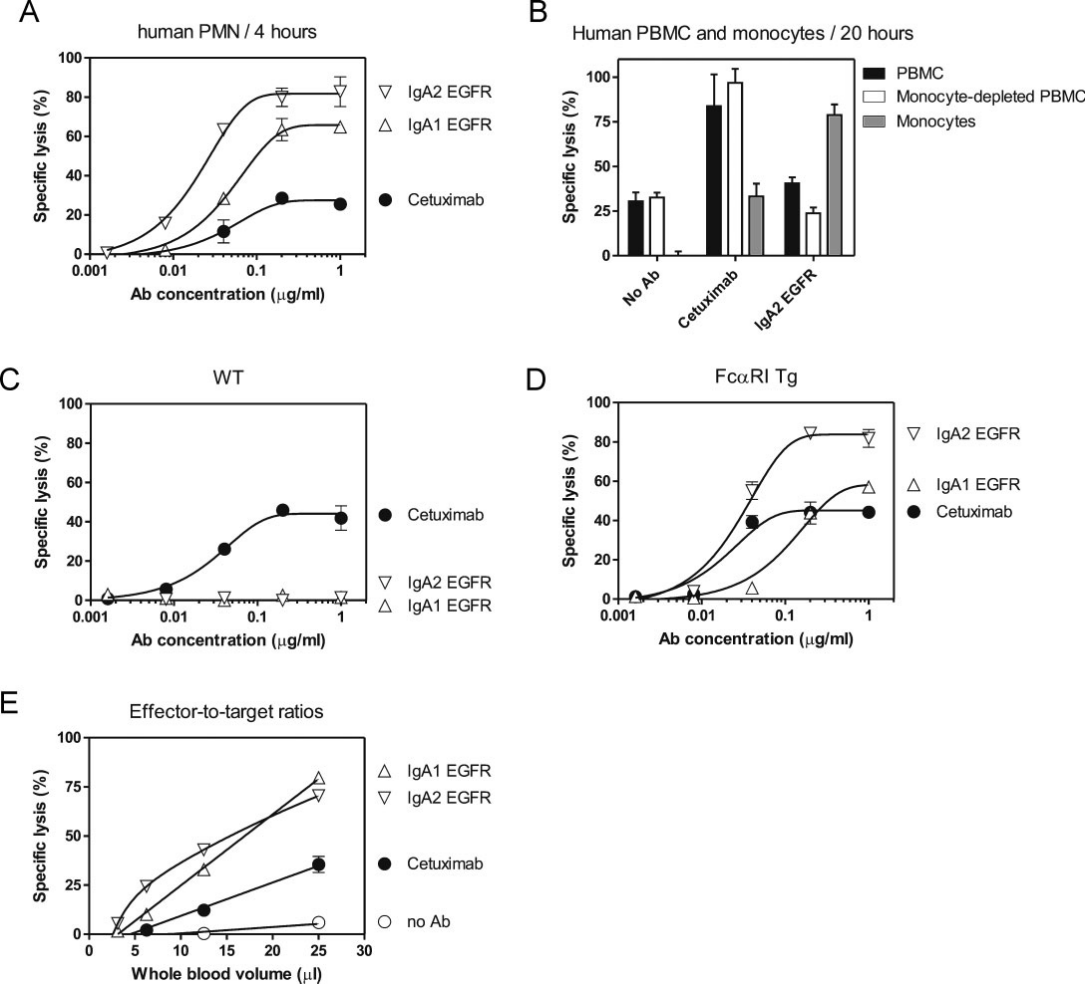
**IgA EGFR mediates efficient tumour cell killing *ex vivo*.**Specific lysis of A1207 cells by isolated human PMNs (E:T = 80:1) in the presence of EGFR antibodies in a 4 h ^51^Cr-release assay.Specific lysis of A1207 cells using human PBMC (E:T = 100:1), monocyte-depleted PBMC fraction (E:T = 100:1) or isolated CD14^+^ monocytes (E:T = 25:1) in a 20 h ^51^Cr-release assay using 1 μg/mL EGFR antibodies.Specific lysis of human A1207 cells *in vitro* by G-CSF-primed whole mouse blood using wild-type C57BL/6 (C) or FcαRI Tg (D) mice in a 4 h ^51^Cr-release assay.Specific lysis of A1207 cells at different E:T ratios with 10 μg/mL EGFR antibody using G-CSF-primed whole blood from FcαRI Tg mice. Data are presented as mean percentage of specific lysis ± SEM from one representative experiment out of three using pooled mouse blood.

To assess the anti-tumour activity of IgA antibodies we previously generated IgA1 and IgA2 variants of the human IgG1 EGFR antibody cetuximab (c225; Dechant et al, 2007; Lohse et al, 2011). IgA1 and IgA2 EGFR have similar target affinity and are as potent as cetuximab in eliciting Fab-mediated anti-tumour effects, such as inhibition of tumour growth and internalization of EGFR (Dechant et al, 2007). In line with previous findings, both IgA1 and IgA2 EGFR exhibited stronger ADCC activity than cetuximab using human PMNs or whole blood as effectors against A1207 cells (Dechant et al, 2007; Peipp et al, 2008b) (Fig 1 and unpublished data). Tumour lysis by peripheral blood mononuclear cells (PBMCs) was only efficient with cetuximab and was likely mediated by NK cells (Supporting Information Fig S1A). Isolated CD14-positive monocytes did not induce tumour lysis in a 4 h assay (Supporting Information Fig S1B). These results show that PMNs are the only blood-resident effector cells that mediate IgA EGFR activity in 4 h assays. However, when monocytes were used in an overnight assay, marked tumour lysis was detected, which was higher by IgA2 EGFR than by cetuximab (Fig 1). This is in line with a recent report showing that monocytes/macrophages can mediate significant tumour cell killing with IgA EGFR (Lohse et al, 2012).

To study whether human IgA EGFR mediate anti-tumour activity by mouse effector cells *ex vivo* we used whole blood from G-CSF-stimulated FcαRI Tg and WT control mice as effector cells. Blood from these mice typically contained ∼50% of PMNs and ∼10% monocytes (Supporting Information Fig S2A). Both PMNs and monocytes expressed mouse FcγRs and the human FcαRI (Supporting Information Fig S2B), however, it is likely that tumour cell killing by whole blood is primarily mediated by PMNs since they are more abundant. It is important to note that in contrast to human PMNs, mouse PMNs do not express FcγRIa or FcγRIIIb (Biburger et al, 2011). We used human A1207 cells expressing high levels of EGFR as target cells. WT PMNs were able to lyse target cells at low concentrations of cetuximab (Fig 1). FcαRI Tg PMNs also lysed tumour cells with both IgA1 and IgA2 EGFR, however, lysis induced by IgA2 EGFR was more efficient (Fig 1). Importantly, the anti-tumour activity of both IgA1 and IgA2 EGFR *ex vivo* was fully dependent on the presence of FcαRI Tg. Tumour cell lysis by both cetuximab and IgA EGFR was already detectable after 120 min (unpublished data). IgA2 EGFR seemed to be more efficient at lower effector-to-target (E:T) ratios (Fig 1).

Taken together, we confirmed that IgA EGFR is more efficient than cetuximab in tumour cell killing by human myeloid effector cells. In addition, we showed that human IgA EGFR mediates efficient cytotoxicity *ex vivo* by mouse effector cells expressing human FcαRI.

### Human IgA EGFR antibodies have a short serum half-life in mice

**Figure 2:**
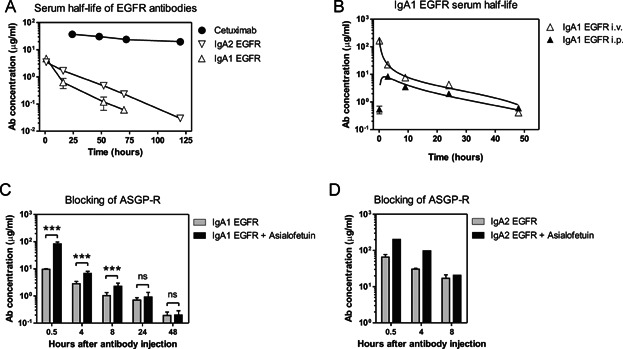
**Serum half-life of human IgA EGFR in SCID mice**SCID mice were injected intraperitoneally with 100 μg of IgA1 EGFR, IgA2 EGFR or cetuximab. Blood was drawn at the indicated time points and the concentration of circulating antibodies was determined by ELISA. The first time point represents 1 h.Serum levels of circulating IgA1 EGFR after intraperitoneal or intravenous injection of antibodies. The first time point represents 5 min.Blocking asialoglycoprotein receptor (ASGP-R) by the injection of 3 mg asialofetuin on Day 0 temporarily inhibited the clearance of i.v. injected 200 μg IgA1 and IgA2 EGFR *in vivo* in BALB/c mice (***p < 0.001 Students' *t*-test).

IgG exhibits a long serum half-life through protection against catabolism by FcRn (Junghans & Anderson, 1996). Since IgA do not bind to FcRn it has a much shorter half-life, which is estimated to be approximately 3–6 days in humans (Monteiro & van de Winkel, 2003). To determine the serum half-life of human IgA EGFR in SCID mice we injected 100 μg IgA1 or IgA2 EGFR i.v. or i.p. and measured serum antibody concentration at the indicated time points (Fig 2). IgA serum half-life was not influenced by the genetic background of the mice (C57BL/6, BALB/c, SCID) or by the presence of the FcαRI Tg (unpublished data). Elimination curves of human IgA EGFR in the mouse were seemingly characterized by two exponential components. In the initial fast component, which occurred within the first hours after intravenous injection, serum IgA concentrations dropped substantially (distribution phase). This is — at least partly — caused by equilibration between blood and the extravascular space. Based on the slower second component the elimination half-life of both IgA1 and IgA2 EGFR was estimated to be approximately 15 h (Supporting Information Table S1). In contrast, the half-life of human IgG1 was at least 4 days, in line with published data (Junghans & Anderson, 1996; Sell & Fahey, 1964).

Human serum IgA has a complex glycosylation pattern, including the presence of terminal sialic acids (Woof & Kerr, 2006). Studies in rats showed that human IgA is primarily cleared from the circulation by receptors recognizing asialated proteins (Bogers et al, 1989; Rifai et al, 2000). In humans, asialoglycoprotein receptor (ASGP-R) is proposed to be involved in the clearance of IgA in blood (Stockert et al, 1982). Blocking ASGP-R by injection of asialofetuin prior to injection of IgA1 or IgA2 EGFR inhibited clearance of IgA EGFR from the serum in the first hours, indicating that clearance by glycoprotein receptors, such as ASGP-R, contributes to the short serum half-life of human IgA EGFR in mice (Fig 2). IgA1 EGFR appeared to be more susceptible than IgA2 EGFR to this clearance mechanism, as suggested by the faster decrease in serum concentration during the first hours. To investigate this we analysed the glycosylation of the IgA antibodies.

*N*-glycoprofiling of IgA1 and IgA2 EGFR demonstrated that both antibodies had different glycosylation (Supporting Information Table S2 and Fig S3). The most striking difference was that about 20% of the glycans from IgA2 EGFR were sialylated, whereas no sialylated glycans were detected for IgA1 EGFR. Although both antibodies had similar neutral *N*-glycan structures (*i.e*. without sialic acids), the IgA1 EGFR also had less Man5 and slightly more mono and bi-antennary glycans than the IgA2 EGFR. As the presence of sialic acid is known to prolong serum half-life by protection from clearance by ASGP-R, and that the addition of asialofetuin prior to injection of IgA1 or IgA2 EGFR inhibited clearance of IgA EGFR from the serum in the first hours, then we can conclude that the sialylation on IgA2 EGFR is the most critical factor for prolonged serum half-life.

Taken together, human IgA EGFR has a substantially shorter serum half-life in mice than human IgG EGFR. This is partially caused by clearance by receptors recognizing incompletely glycosylated proteins.

### Anti-tumour activity of IgA2 EGFR in a xenograft lung model with A431-luc2 cells

IgA2 EGFR showed stronger ADCC activity than IgA1 EGFR *in vitro* with both human and mouse effector cells (Fig 1; Dechant et al, 2007). In addition, IgA2 EGFR exhibited more favourable *in vivo* kinetics; showing smaller decrease during the distribution phase. Therefore in subsequent *in vivo* studies we used IgA2 EGFR to assess the anti-tumour activity of IgA antibodies.

*In vivo* activity of IgA2 EGFR was tested in a long-term xenograft tumour model, using the human epidermoid cell line A431, transduced with a luciferase gene (A431-luc2) allowing longitudinal monitoring of tumour growth by bioluminescent imaging (BLI). A431 cells endogenously express high levels of EGFR (although lower than A1207 cells) and are partially dependent on EGFR signalling for proliferation. We used human FcαRI Tg SCID mice expressing human FcαRI in a physiologically relevant pattern (van Egmond et al, 1999).

**Figure 3:**
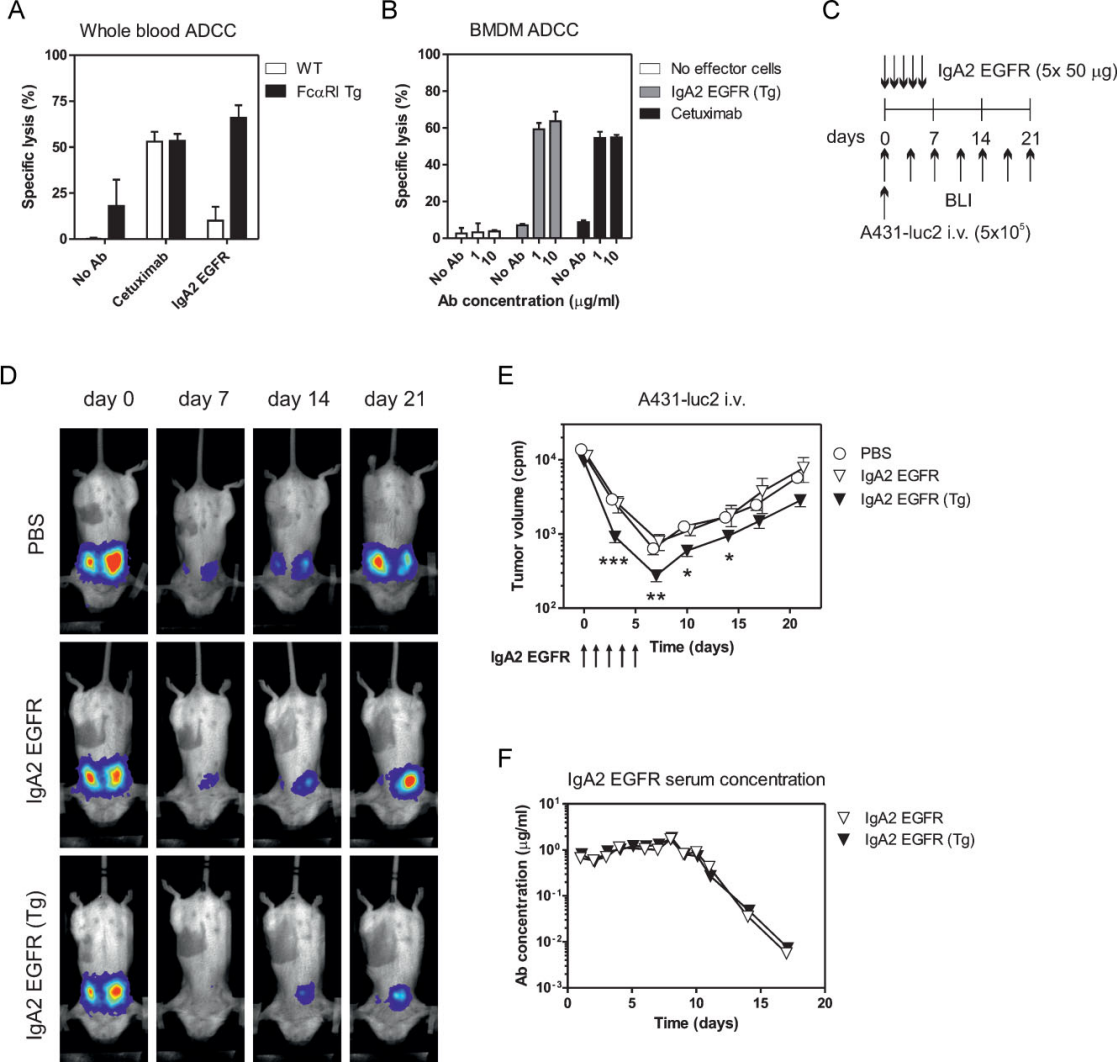
**Anti-tumour activity of IgA2 EGFR in an intravenous xenograft model**Specific lysis of A431-luc2 cells *in vitro* by G-CSF-primed mouse whole blood with 1 μg/mL EGFR antibodies in a 4 h ^51^Cr-release assay.Specific lysis of A431 cells by mouse bone marrow macrophages with EGFR antibodies in an overnight ^51^Cr-release assay.Scheme of A431-luc2 i.v. model. 5 × 10^5^ A431-luc2 cells were injected intravenously into FcαRI Tg SCID or WT SCID mice. Mice were injected i.p. with 50 μg IgA2 EGFR daily until Day 4. Tumour growth was monitored by serial BLI.Representative images from the BLI recordings of the A431-luc2 i.v. experiment.The curves represent the mean tumour volume (cpm) ± SEM for each measurement (seven mice/group, *p < 0.05, **p < 0.01, ***p < 0.001; Student's test, significance was assessed compared to the PBS group).Concentrations of IgA2 EGFR in serum during the A431-luc2 i.v. experiment. At each time point one mouse was bled from FcαRI Tg and WT groups.

Both cetuximab and IgA2 EGFR were previously shown to induce growth inhibition of A431 cells *in vitro* (Dechant et al, 2007). Both G-CSF-stimulated whole mouse blood, mainly containing PMNs (Fig 3), and macrophages (Fig 3) were able to lyse A431 cells *in vitro*. This suggests that both PMNs and macrophages could contribute to the cytotoxic activity of EGFR antibodies *in vivo*.

We injected 5 × 10^5^ A431-luc2 cells intravenously into FcαRI Tg or WT littermate SCID mice. In the first 4 days the mice were treated intraperitoneally with 50 μg IgA2 EGFR daily, and tumour growth was followed by serial BLI (Fig 3). Detectable tumour outgrowth was restricted to the lungs (Fig 3). In this model, the strong decrease in lung BLI signal between Day 0 and 3 in PBS-treated mice suggest that the majority of the injected A431-luc2 cells either die or migrate to deep tissues, where less BLI signal can be detected. All mice were injected with recombinant G-CSF as it was shown to enhance tumour cell killing by FcαRI (Stockmeyer et al, 2000). IgA2 EGFR significantly decreased tumour growth until Day 14 in FcαRI Tg but not in WT SCID mice (Fig 3). At later time points, this treatment effect was no longer significant, probably due to the sharp decline in serum IgA2 EGFR concentrations (Fig 3).

From these experiments we concluded that IgA2 EGFR elicits *in vivo* anti-tumour activity that was strictly dependent on the presence of FcαRI, indicating that in this model system Fc-independent effector mechanisms did not contribute to the observed effects.

### Anti-tumour activity of IgA2 EGFR in a xenograft peritoneal model with A431-luc2 cells

**Figure 4:**
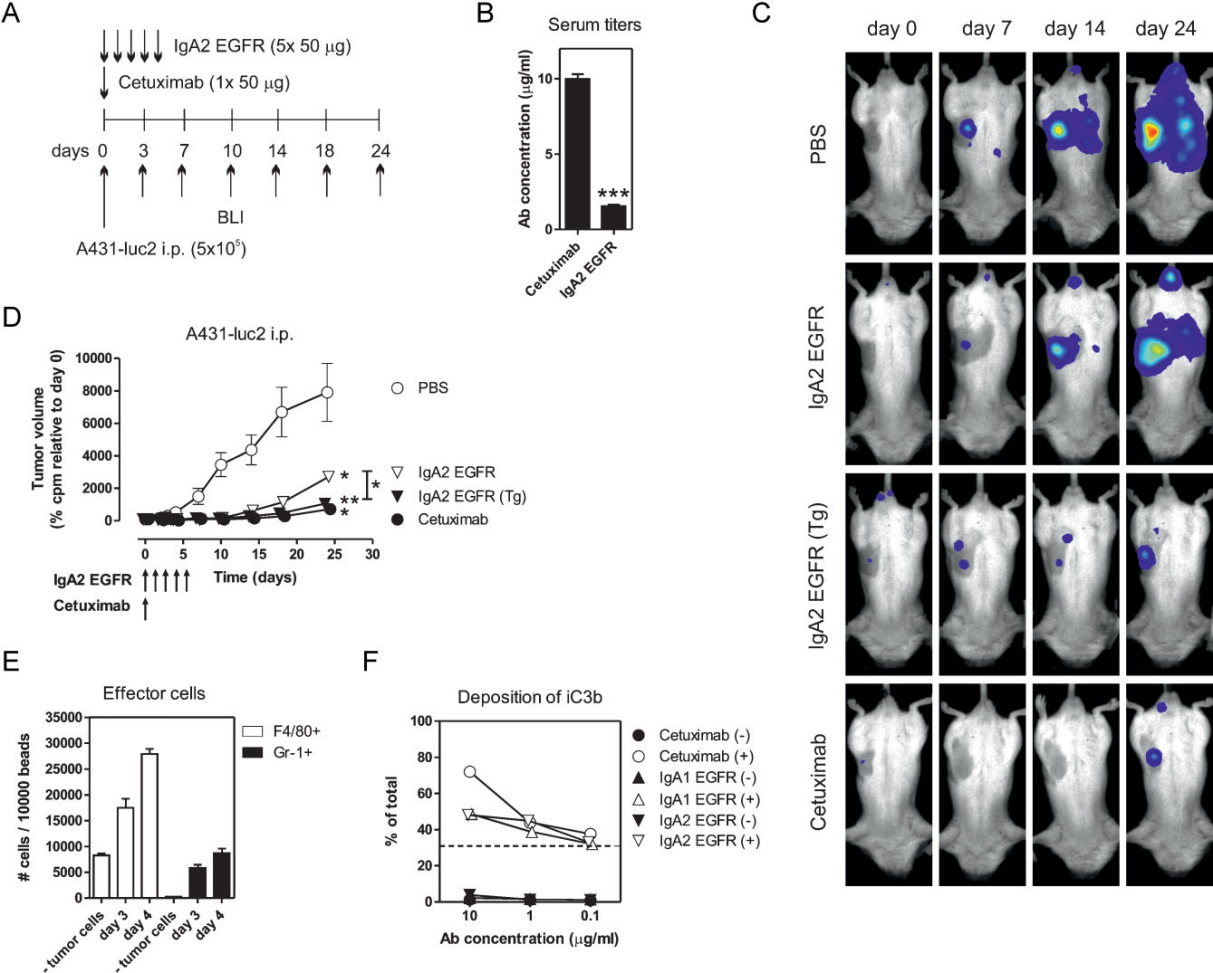
**Anti-tumour activity of IgA2 EGFR in a peritoneal xenograft model**A431-luc2 i.p. model. FcαRI Tg SCID or WT SCID mice were injected with 5 × 10^5^ A431-luc2 cells intraperitoneally with 50 μg cetuximab once on Day 0, or with 50 μg IgA2 EGFR daily until Day 4. Tumour growth was monitored by serial BLI.Average serum concentration of IgA2 EGFR and cetuximab during the first 5 days of the A431-luc2 i.p. experiment.Representative BLI images of the A431-luc2 i.p. experiment.Tumour growth as detected by BLI (counts per minute, cpm). Data are pooled from two experiments and expressed as relative BLI signal compared to the signal recorded on Day 0 (*p < 0.05, **p < 0.01, Mann–Whitney test, 5–10 mice/group, significance was assessed compared to the PBS-treated group).Effector cells in the peritoneum. Effector cells were identified by FACS in peritoneal lavage on Day 3 and on Day 4 after the injection of A431-luc2 cells. Their relative number was compared using known amount of beads.Deposition of complement fragments *in vitro*. A431 cells were opsonized with 1 μg/mL EGFR antibodies for 30 min at room temperature and incubated with (open symbols) or without (closed symbols) complement-active mouse serum for 45 min at 37°C. Deposited complement fragments were detected by specific antibody against mouse C3b/iC3b/C3c.

Next, we used the A431-luc2 cells in a peritoneal model. In this model higher antibody concentrations were expected to reach the tumour environment, because EGFR antibodies were also injected intraperitoneally. In contrast to the lung model, tumour cells in PBS-treated mice showed direct outgrowth after injection. EGFR antibody was administered once on Day 0 (50 μg cetuximab) or daily until Day 4 (50 μg IgA2 EGFR) to compensate for the shorter half-life of IgA2 (Fig 4). The concentrations of circulating IgA2 EGFR during the first 5 days of the experiment were approximately five times lower than those of cetuximab (Fig 4). Despite this difference, cetuximab and IgA2 EGFR were both effective in suppressing tumour outgrowth in FcαRI Tg mice (Fig 4). IgA2 EGFR was also effective in WT SCID mice indicating that its anti-tumour effects in this model were largely independent of FcαRI Tg, suggesting the involvement of Fab-mediated anti-tumour effects (Fig 4). Nevertheless, IgA2 EGFR was more effective in FcαRI Tg than in WT mice, indicating the contribution of Fc-mediated effector mechanisms to the anti-tumour effects. Injection of tumour cells increased the number of peritoneal macrophages and PMNs in the peritoneum (Fig 4) and they both expressed human FcαRI (unpublished data). Therefore both PMNs and macrophages could contribute *in vivo* to the ADCC mediated by IgA2 EGFR.

Conventional IgG1 EGFR antibodies against a single EGFR epitope cannot induce CDC of opsonized A431 cells (Dechant et al, 2008). When IgA EGFR was added to combinations of IgG EGFR, CDC was inhibited (Lohse et al, 2010). However, IgA EGFR may activate complement via the lectin or the alternative pathway, which could lead to deposition of complement fragments, such as iC3b, on tumour cells. *In vitro* incubation of IgA EGFR-opsonized A431 cells with complement active mouse blood resulted in deposition of iC3b on tumour cells, albeit less than what is induced by cetuximab (Fig 4). These fragments may engage complement receptors, such as CR3, expressed on effector cells and thereby enhance tumour cell killing as seen in CD20 therapy (Boross et al, 2011; van Spriel et al, 2003).

Collectively, these data obtained using two xenograft models show effective anti-tumour activity by IgA2 EGFR *in vivo* against a tumour cell line with endogenous EGFR expression at two different anatomical locations. The mechanisms of action of EGFR antibodies against A431 cells *in vivo* are likely to involve a combination of Fab- and Fc-mediated effects depending on the antibody concentration at the tumour site.

### IgA2 EGFR induces cytotoxicity of EGFR-expressing Ba/F3 cells in a syngeneic intraperitoneal model

**Figure 5:**
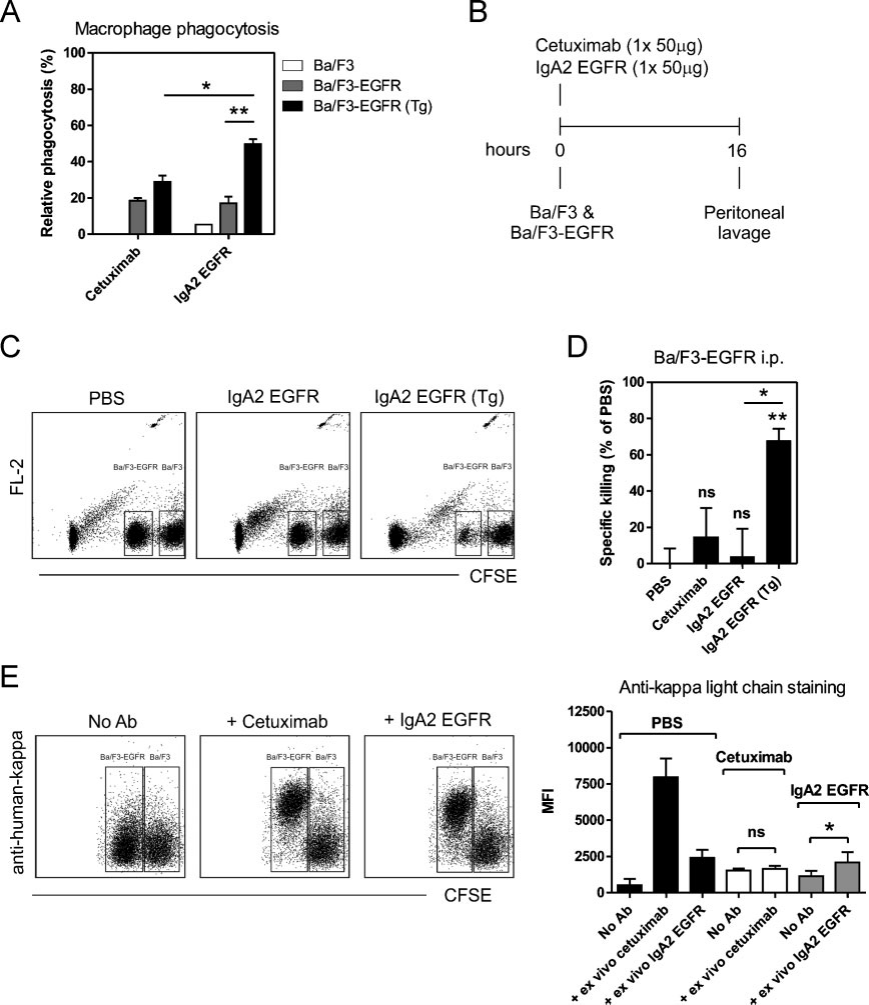
**Anti-tumour activity of IgA2 EGFR in a syngeneic peritoneal model using human EGFR-transfected Ba/F3 cells***In vitro* assay to determine elimination of Ba/F3-EGFR cells by macrophages. Bone marrow-derived macrophages from WT and FcαRI Tg BALB/c mice were incubated overnight with CFSE-labelled Ba/F3-EGFR cells at E:T ratio of 10:1 in the presence of 1 μg/mL EGFR antibodies. At the end of the incubation, cells were transferred into a new tube and the number CFSE-positive cells was determined. In wells with EGFR mAbs the number of Ba/F3-EGFR cells was decreased. Cytotoxicity of Ba/F3-EGFR cells was calculated by comparing this decrease in Ba/F3-EGFR numbers compared to PBS-conditions (*p < 0.05, **p < 0.01; Student's *t*-test).Ba/F3-EGFR i.p. model. We injected a 1:1 mixture of Ba/F3 and Ba/F3-EGFR cells (10^7^ cells in total) differentially labelled with high and low concentrations of CFSE into the peritoneum of FcαRI Tg or WT BALB/c mice. Immediately after the injection of the cells, the mice received 50 μg cetuximab or IgA2 EGFR. After 16 h the peritoneum was washed and the ratio of Ba/F3 and Ba/F3-EGFR cells was analysed to calculate specific cytotoxicity.Representative dot plots showing cells retrieved from the peritoneum. Ba/F3 and Ba/F3-EGFR cells can be identified based on their CFSE-labelling. Dot plot are shown from PBS-treated mice, IgA2 EGFR-treated WT and FcαRI Tg mice. TruCount beads (visible in the upper right corner) were used to calculate the absolute numbers of cells.*In vivo* cytotoxicity by EGFR antibodies in the Ba/F3-EGFR i.p. model. Specific cytotoxicity is calculated from ratio of Ba/F3 and Ba/F3-EGFR cells in each treatment group. The change in this ratio was compared to PBS-treated mice (*p < 0.05; **p < 0.01; ANOVA, Bonferroni post test, 4–10 mice/group). Representative results of three independent experiments.*Ex vivo* analysis of target opsonization by cetuximab or IgA2 EGFR. Ba/F3-EGFR cells recovered from the peritoneum were identified by CFSE-labelling. Bound EGFR antibodies on the tumour cells were revealed by *ex vivo* staining with anti-human kappa-light-chain antibodies. To assess the total number of available EGFR molecules Ba/F3-EGFR cells were incubated with extra EGFR antibodies *ex vivo*. In case of complete target opsonization this did not increase the signal (cetuximab). An increase, as seen in IgA2 EGFR group indicates that not all EGFR molecules were opsonized *in vivo*. Representative FACS plot and graph with mean fluorescent intensity of five mice/group is shown (*p < 0.05; Student's *t*-test).

To study cytotoxic activity of IgA2 EGFR in syngeneic setting we used human EGFR-transfected Ba/F3 cells (Ba/F3-EGFR) expressing lower levels of EGFR *in vitro* than A431 cells (Peipp et al, 2008c). Ba/F3-EGFR cells could not be lysed *ex vivo* by G-CSF-primed mouse whole blood (unpublished data). However, FcαRI Tg mouse macrophages efficiently phagocytosed Ba/F3-EGFR cells in the presence of IgA2 EGFR (Fig 5). Phagocytosis of untransfected Ba/F3 cells or of Ba/F3-EGFR cells in the presence of cetuximab or control IgG or IgA antibodies was less efficient (Fig 5 and unpublished data).

We used Ba/F3-EGFR cells in a short, syngeneic peritoneal model (Fig 5; Boross et al, 2011; de Haij et al, 2010). This model enables the exact analysis of a number of parameters, such as tumour cell numbers, E:T ratios, target expression and opsonization efficacy. FcαRI Tg or WT BALB/c mice were injected intraperitoneally with a 1:1 mixture Ba/F3 and Ba/F3-EGFR cells (in total 5 × 10^6^ cells) differentially labelled with CFSE. Mice were treated with 50 μg of EGFR antibodies immediately after the injection of Ba/F3 cells. The peritoneum was washed 16 h later and the ratio of Ba/F3 and Ba/F3-EGFR cells was determined by FACS analysis to calculate the specific cytotoxicity (Fig 5). In the presence of mIL-3, Ba/F3-EGFR cells are not dependent on EGF signalling for survival *in vitro* (Peipp et al, 2008c). In line with this, the number of Ba/F3-EGFR cells we recovered from the peritoneum after 16 h was similar to the injected amount (unpublished data).

Injection of cetuximab did not induce significant killing of Ba/F3-EGFR cells (Fig 5). As expected, IgA2 EGFR was not effective in WT mice, because Ba/F3-EGFR cells are not susceptible to direct growth inhibition by EGFR antibodies under these conditions. However, significant cytotoxicity was seen with IgA2 EGFR in FcαRI Tg mice (Fig 5).

Ba/F3-EGFR cells upregulated EGFR expression *in vivo* compared to *in vitro* cultured cells (unpublished data). To determine whether EGFR antibodies had fully opsonized their target, we analysed target binding on the tumour cells recovered from the peritoneum identified by their CFSE signal (Fig 5). We stained tumour cell-bound EGFR antibodies using anti-kappa-light chain antibodies, with or without addition of extra EGFR antibodies *ex vivo*. In cetuximab-treated mice, no extra signal was detected after adding extra cetuximab *ex vivo*, indicating that all EGFR molecules were occupied. In contrast, EGFR was not fully opsonized by IgA2 EGFR (Fig 5).

In conclusion, IgA2 EGFR mediated efficient *in vivo* cytotoxicity of Ba/F3 cells expressing EGFR in a short peritoneal model, which was fully dependent on the presence of FcαRI.

### *In vivo* IgA2 EGFR activity is mediated by macrophages

**Figure 6:**
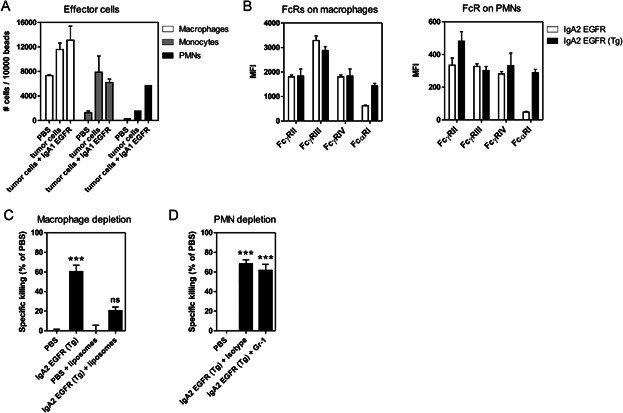
***In vivo* IgA2 EGFR activity is mediated by macrophages in a syngeneic peritoneal model using Ba/F3-EGFR cells**Effector cells in the peritoneum in the Ba/F3-EGFR i.p. model. Effector cell types were identified by FACS and their relative number was determined compared to known amount of beads.Expression of FcRs on the different effector cell types in the peritoneal lavage during the Ba/F3-EGFR i.p. model in PBS-treated mice. Expression of mouse FcγRs and human FcαRI on macrophages (F4/80^+^) and PMNs (Ly6G^high^) was analysed by FACS.Depletion of macrophages in the Ba/F3-EGFR i.p. model. Specific cytotoxicity of Ba/F3-EGFR cells in FcαRI Tg mice treated with 50 μg IgA2 EGFR or PBS. Macrophages were depleted prior to the experiment by injection of chlodronate liposomes (***p < 0.001, ANOVA, Bonferroni post test, four to five mice/group).Depletion of PMNs in the Ba/F3-EGFR i.p. model. Specific cytotoxicity of Ba/F3-EGFR cells in FcαRI Tg mice treated with 50 μg IgA2 EGFR or PBS. Prior to the experiment, mice were injected with 250 μg Gr-1 (Ly6G/C-specific) antibody, or with isotype control, two times intraperitoneally to deplete PMNs (***p < 0.001, ANOVA, Bonferroni post test, 9–10 mice/group).

The injection of Ba/F3 cells increased the numbers of effector cells in the peritoneum, including macrophages (F4/80^+^), resident monocytes (Ly6G^low^) and PMNs (Ly6G^high^) (Fig 6). Both macrophages and PMNs expressed mouse FcγRs and human FcαRI (Fig 6). Recruitment of effector cells or expression of mouse FcγR was the same for both WT and FcαRI Tg mice (unpublished data). Expression of FcγR was higher on macrophages than on PMNs and was not influenced by the antibody treatment (Fig 6 and unpublished data). To identify the effector cells are responsible for the cytotoxicity by IgA2 EGFR, PMNs or macrophages were selectively depleted by injection of depleting antibodies or chlodronate liposomes, respectively. Depletion was specific and its efficacy was over 99% for PMNs (Supporting Information Fig S4A) and over 90% for macrophages (Supporting Information Fig S4B). The *in vivo* activity of IgA2 EGFR was fully abrogated by the depletion of macrophages (Fig 6), but not by the depletion of PMNs (Fig 6).

These results show that macrophages are the predominant effector cells in this model.

### IgA2 EGFR elicits anti-tumour activity in an immunocompetent lung metastasis model with B16F10-luc2-EGFR cells

To test the anti-tumour activity of IgA EGFR in an immunocompetent tumour model we used the well-established lung metastasis model with B16F10 cells (de Haij et al, 2010). We set up B16F10-luc2-EGFR cells that grew reproducibly in WT immunocompetent C57BL/6 mice.

**Figure 7:**
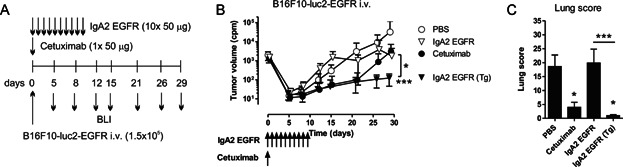
**Anti-tumour activity of IgA2 EGFR in an immunocompetent model**B16F10-luc2-EGFR i.v. model. FcαRI Tg or WT C57BL/6 mice were injected with 1.5 × 10^5^ B16F10-luc2-EGFR cells intravenously. Mice were treated with 50 μg cetuximab once on Day 0, or with 10 × 50 μg IgA2 EGFR daily until Day 9. Tumour growth was monitored by serial BLI.The curves represent mean tumour volume as determined by bioluminescent imaging (counts per minute, cpm) ± SEM (6–10 mice/group, *p < 0.05, ***p < 0.001; Mann–Whitney test, significance was assessed compared to the PBS group).Lung scores determined by visual scoring of the number and size of metastases in the lungs (mean ± SEM, 6–10 mice/group, *p < 0.05, ***p < 0.001; Mann–Whitney test).

We injected 1.5 × 10^5^ B16F10-luc2-EGFR cells intravenously into FcαRI Tg or WT C57BL/6 mice. EGFR antibodies were administered intraperitoneally either once on day 0 (50 μg cetuximab) or daily until Day 9 (10 × 50 μg IgA2 EGFR) to compensate for the shorter half-life of IgA2 EGFR (Fig 7). Tumour cell growth was monitored by serial BLI. At the end of the experiment lungs were removed and scored for the numbers of metastasis.

Injection of cetuximab largely inhibited growth of B16F10-luc2-EGFR cells *in vivo* until Day 20, after which tumour cells started to grow. IgA2 EGFR did not have significant anti-tumour effect in WT mice; however it significantly inhibited tumour growth in FcαRI Tg mice at the end of the experiment compared to controls (Fig 7). Interestingly, the effect of IgA EGFR seemed to last at least a week longer than that of cetuximab, which is surprising, since at Day 21 no circulating IgA2 EGFR could be measured in the serum (unpublished data). In line with the BLI data, cetuximab and IgA2 EGFR also significantly decreased the number of metastasis in the lungs (Fig 7).

These results demonstrated that in the presence of FcαRI, IgA2 EGFR exhibit a potent cytotoxic activity against B16F10-luc2-EGFR cells in a long-term model using immunocompetent mice in a physiologically relevant anatomical location.

## DISCUSSION

Exploiting the cytotoxic functions of FcαRI is a promising strategy for effective immunotherapy (Valerius et al, 1997). Several studies have demonstrated that IgA EGFR mediate potent anti-tumour effects *in vitro* (Beyer et al, 2009; Dechant et al, 2007). Here we provide the first evidence that IgA2 EGFR can mediate potent anti-tumour effects *in vivo*.

EGFR antibodies elicit both Fab- and Fc-mediated anti-tumour effects (Peipp et al, 2008a). We used A431 cells that partially rely on active signalling via EGFR for growth (Dechant et al, 2007), and Ba/F3-EGFR cells, that in the presence of mouse IL-3, are not susceptible to Fab-mediated effector mechanisms, allowing us to focus on Fc-mediated effector functions. The anti-tumour activity of IgA2 EGFR was dependent on FcαRI in both lung models and in the Ba/F3-EGFR peritoneal model, demonstrating a dominant role for ADCC. Interestingly, IgA2 EGFR induced significant anti-tumour activity also in WT SCID mice in the A431 peritoneal model, indicating a contribution of Fab-mediated mechanisms to the anti-tumour effects. It was previously shown that IgG1 EGFR antibodies have a dual mode of action: at high antibody concentration the anti-tumour effect is primarily achieved by growth inhibition, whereas at lower antibody concentration they act via ADCC (Bleeker et al, 2004). It is likely that, in contrast to the A431 lung model, in the A431 peritoneal model higher antibody concentrations were achieved that were sufficient for effective Fab-mediated effects.

The anti-tumour activity of IgG is regulated by the balance of activating and inhibiting FcγRs (Clynes et al, 2000). Human IgG1 is known to interact well with mouse FcγR (Overdijk et al, 2012). Therefore it was surprising that in the Ba/F3-EGFR peritoneal model, despite complete target opsonization, cetuximab was not efficient. However, cetuximab mediated significant cytotoxicity against five times less Ba/F3 cells indicating that cetuximab is able to induce cytotoxic activity in this model when the E:T ratios are more favourable (unpublished data). This is in line with the *in vitro* data showing that IgA2 EGFR is more efficient at lower E:T ratios.

Previous studies using human effector cells showed that IgA anti-tumour antibodies effectively recruit blood PMNs to mediate tumour-killing (Dechant et al, 2007). Our results *in vitro* with mouse whole blood enriched for PMNs using an adherent tumour cell line confirm this. Interestingly, we found that PMNs did not contribute to the killing of the non-adherent Ba/F3-EGFR cells both *in vitro* and *in vivo*, but that these cells are primarily eliminated by macrophages. Differentiated mouse macrophages or isolated human blood monocytes were able to lyse A431 cells in an overnight killing assay. More recently, efficient tumour cell killing by IgA EGFR by differentiated human macrophages was demonstrated even at low E:T ratios (Lohse et al, 2012). This shows that, depending on the target cell line, both PMNs and monocytes/macrophages can mediate cytotoxicity by IgA anti-tumour antibodies.

Macrophages, NK cells and PMNs use different mechanisms for tumour cell killing. Macrophages seem to rely more on phagocytosis, independent of the type of antibodies used. Ba/F3-EGFR cells are smaller than A431 cells and thus may be phagocytosed more efficiently. NK cells elicit tumour cell lysis by IgG by inducing apoptosis in the target cell through the release of perforins and granzymes. PMNs are recently described to induce autophagy in the target cell when IgA is used (Bakema et al, 2011). In addition, PMNs can also eliminate tumour cells via frustrated phagocytosis or reversed endocytosis (Cordier et al, 1981). The precise contribution of these mechanisms to the tumour cell killing induced by IgG or IgA antibodies should be further investigated. In addition, tumour cells often overexpress CD47, which — via interaction with signal-regulatory protein-alpha (SIRPα) — inhibits elimination of tumour cells by macrophages (Chao et al, 2012). Tumour-associated macrophages found in many solid tumours may be converted into tumour-surveillance cells after blocking this interaction.

It is evident that the serum half-life of IgA *in vivo* was substantially shorter than that of IgG. Depending on the study design and the antibody used, the half-life of mouse or human IgA in mice was estimated to be in the range of 12–48 h using radioactive labelling (Rifai & Mannik, 1983; Sancho et al, 1984). Our result of 15 h which was obtained using ELISA, a technique that only detects intact antibodies, was within this range. Even though the half-life of IgA in humans is most likely longer, in the range of 5 days, it is still substantially shorter than that of IgG (Monteiro & van de Winkel, 2003). Because of this difference, it is very difficult to directly compare efficacy of IgG and IgA *in vivo* using similar antibody conditions in the tumour environment. Our data with the short peritoneal Ba/F3-EGFR model suggest that even after injection of equal amounts of antibody, target opsonization by IgA2 EGFR was not complete. In the A431 peritoneal model, despite injection of more IgA2 EGFR, circulating IgA2 EGFR levels were at least five times lower during the first 5 days of the experiment. This strongly suggest that opsonization by IgA2 EGFR was not complete *in vivo*. Despite these suboptimal conditions IgA2 EGFR elicited significant anti-tumour activity.

Fast initial clearance causes a steep decline in serum IgA levels, which is most likely a consequence of uptake by receptors recognizing incomplete glycosylation, since blocking or genetic deletion of ASGP-R significantly enhanced IgA serum levels (Bogers et al, 1989; Rifai et al, 2000). The glycosylation of IgA is more complex than that of IgG (Woof & Kerr, 2006). Moreover, approximately 10% of serum IgA1 also carry *O*-linked glycosylation in the hinge region (Tarelli et al, 2004). Therefore, better understanding of IgA glycosylation could lead to extended serum half-life of IgA through glycoengineering. Importantly, despite the shorter half-life and incomplete opsonization, IgA2 EGFR efficiently mediated anti-tumour effects.

IgA may be an alternative antibody format in the future for antibody therapy. FcαRI is expressed differentially on human effector cells and IgA activity is not regulated by FcγRIIb, therefore it is expected that IgA will trigger different effector functions *in vivo* compared to IgG. Because of its physiological functions IgA may be able to reach different anatomical areas than IgG. However potential toxicity of IgA could be a problem, since PMNs, expressing high levels of FcαRI are abundantly present in blood and may be triggered extensively.

These results demonstrate for the first time potent *in vivo* cytotoxic activity of IgA2 EGFR anti-tumour antibodies, suggesting that effective immunotherapy can be achieved by exploiting the cytotoxic functions of FcαRI.

## MATERIALS AND METHODS

### Mice

Human FcαRI transgenic (Tg) mice were generated at the UMC Utrecht (van Egmond et al, 1999), and were backcrossed to C57BL/6, BALB/c and immunodeficient SCID background (CB17/lcr-*Prkdc*^scid^/lcrlcoCrl, Charles River) and maintained as hemizygous. Transgene negative littermates or WT BALB/c or C57BL/6 mice (Janvier) were used as controls. All experiments were approved by the Animal Ethical Committee of the UMC Utrecht.

### Cell lines

A1207 and A431 cell lines (obtained from ATCC) were cultured in RPMI1640 (Gibco) supplemented with 10% FCS, penicillin and streptomycin. A431 cells were derived from a human epidermoid carcinoma and have high surface expression of EGFR. Therefore A431 cells are frequently the target line chosen for evaluation of cytolytic activity and anti-tumour cell activity of a therapeutic agents intended for use against carcinomas. A1207 cells are derived from a glioma, and also carry high levels of EGFR. A431 cells were transduced lentivirally with luciferase–GFP construct; two independent A431-luc2 clones were selected and tested for *in vivo* outgrowth in SCID mice. Ba/F3 cells are bone-marrow-derived, immortalized cells, representing an early pro-B cell stage, which are IL-3-dependent. Ba/F3 cells were cultured in RPMI1640 medium supplemented with 10% FCS, penicillin and streptomycin and mouse IL-3, as described in Bracke et al (2000). Ba/F3 cells were transfected with WT EGFR (Upstate) and EGFR expressing clones were selected using neomycin (Peipp et al, 2008c). B16F10 mouse melanoma cells were transfected with the human EGFR gene. A clone was established that expressed stable levels of EGFR *in vitro* and grew reproducibly in WT immunocompetent C57BL/6 mice.

### Antibodies and flow cytofluorimetry

The chimeric anti-human EGFR antibody cetuximab (Erbitux, Merck KGaA) was purchased from the pharmacy of UMC Utrecht. The generation and characterization of IgA EGFR was previously described (Beyer et al, 2009; Dechant et al, 2007). *N*-glycoprofiling of IgA1 EGFR and IgA2 EGFR was performed as described earlier (Royle et al, 2003, 2008). As secondary antibody anti-human-IgG (Caltag) or biotin-labelled goat F(ab')2 anti-human kappa (SouthernBiotech) was used. Expression of FcγRI was determined using anti-mouse CD64 (X54-5/7.1.1, BD Pharmingen). Expression of FcγRIII was determined by blocking the cells with 5 μg/mL FcγRIIB-specific mAb (clone K391, Ly17.2-specific) followed by staining with anti-FcγRII/III (2.4G2, BD Pharmingen). The FcγRIV-specific mAb (9E9) was a gift from Prof. Falk Nimmerjahn (Erlangen). Expression of FcαRI was determined using anti-human CD89 (A59, BD Pharmingen). Expression of FcγRs and FcαRI by effector cells was determined on the peritoneal lavage or on primary bone marrow-derived macrophages after blocking with 5% mouse serum. For staining of cells in the blood 15 μL of whole mouse blood was stained at room temperature. The following antibodies were used for the identification of the different effector cell types: F4/80 (BM8, Biolegend), CD11b (M1/70, BD Pharmingen), Ly-6G (1A8, Biolegend) and Ly-6G/C (RB6-8C5, eBioscience). Complement deposition was measured using anti-mouse C3b/iC3b/C3c (clone 3/26, Hycult Biotech) followed by secondary polyclonal goat anti-rat Ig (Pharmingen). Cytofluorimetry measurements were performed on FACSCalibur or FACSCantoII (BD Biosciences). Data were analysed by Cell Quest or FACS Diva software.

### ADCC assay

#### Mouse ADCC

The mouse whole blood ADCC assay has been described earlier (de Haij et al, 2010). Briefly, WT or FcαRI Tg mice were injected with 10–20 μg of recombinant human PEG-G-CSF (Amgen) subcutaneously in 100 μL PBS. The percentage of circulating PMNs (identified by forward and side scatter and by CD11b^+^Ly6G^+^ staining) was typically around 50%, expression of FcγR was comparable on FcαRI Tg and WT PMNs. Twenty-five microlitres whole blood (unless stated otherwise) collected into Li-heparine tubes from two mice from the retro-orbital plexus was used in the killing assays with whole mouse blood. Target cells (5000 cells/well) were labelled with 100 μCu ^51^Cr for 2 h at 37°C, washed three times in complete medium. Cells were incubated together for 4 h (unless stated otherwise) in 200 μL RPMI1640 + 10% FCS and ^51^Cr release was measured using a γ-scintillator and expressed as counts per minutes (cpm). Percentage of specific lysis was calculated as follows: (experimental cpm − basal cpm)/(maximal cpm − basal cpm) × 100, with maximal lysis determined in the presence of 5% triton and basal lysis in the absence of antibody and effectors.

#### Human ADCC

ADCC assays using ^51^Cr-labelled target cells were performed as described previously (Dechant et al, 2007). Briefly, Ficoll-Histopaque-separated PBMCs or PMNs, sensitizing antibodies in medium were added to round-bottom microtiter plates (Corning Incorporated). Effector-to-target (E:T) ratios were 80:1 for PMNs or 100:1 for PBMCs. The cells were incubated for 4 h at 37°C. Percentage of specific lysis was calculated as for mouse ADCC. Monocytes were isolated from the PBMC fraction and using CD14 magnetic beads (Miltenyi Bioscience) and used at a E:T ratio of 25:1.

### Macrophage killing assay

Bone marrow-derived macrophages (BMDM) were cultured from WT and FcαRI Tg mice in the presence of 5 ng/mL GM-CSF (Cell Sciences) and culture medium was refreshed on Day 2 and 5 as earlier described (de Haij et al, 2010). Adherent cells were used as BMDM on Day 7–8. BMDM were plated together with 10,000 CFSE-labelled Ba/F3-EGFR cells in the presence of EGFR antibodies at an E:T ratio of 10:1. After overnight incubation, cells were trypsinized and the numbers of CFSE positive cells were determined relative to the constant amount of beads (Molecular Probes, Leiden).

### ELISA

Microplates were coated overnight with 0.5 μg/mL anti-human-kappa antibody (Southern Biotech), blocked with 1% BSA in 0.05% Tween20 in PBS, incubated with sera diluted in 1% BSA in 0.05% Tween20 in PBS with for 1.5 h at room temperature. Anti-IgG-HRP (Jackson) or anti-IgA-HRP detection antibody (Southern Biotech) was used and incubated for 1 h before plates were developed with ABTS and read at 405 nm.

### Complement deposition

A431 cells (10^5^ cells/well) were opsonized with EGFR antibodies in medium for 30 min at room temperature. Unbound antibodies were washed away and complement active mouse serum was added at a final concentration of 5% in medium containing 2 mM MgCl_2_ and 2 mM CaCl_2_. Cells were incubated for 45 min at 37°C, washed and stained with anti-iC3b antibody (Hycult Biotechnology), and with anti-rat IgG as a secondary antibody (Pharmingen).

### *In vivo* serum half life of human IgA

IgA1 EGFR, IgA2 EGFR or cetuximab (100 μg) was injected intravenously or intraperitoneally in WT or FcαRI Tg SCID mice (four mice/group). Blood was collected by cheek pouch after the indicated time points and the concentration of human IgA or IgG in the serum was determined by ELISA. In some experiments 3 mg asialofetuin (Sigma) was injected intraperitonealy shortly before the injection of EGFR antibodies into BALB/c mice.

### Ba/F3 peritoneal model

Ba/F3 and Ba/F3-EGFR cells were labelled with 0.125 μM and 1 μM CFSE (Molecular Probes) for 10 min at 37°C and mixed thereafter at 1:1 ratio. In total 10^7^ cells were injected per mouse intraperitoneally in 200 μL PBS. Cetuximab and IgA2 EGFR were injected directly after injection of tumour cells. Sixteen hours later the mice were euthanized and the peritoneum was washed, and the ratio of target negative (Ba/F3) and target positive (Ba/F3-EGFR) cells was determined by cytofluorimetry. Bound EGFR antibodies on the tumour cells were revealed by staining using an anti-kappa antibody (SBA). To detect free EGFR, tumour cells were *ex vivo* incubated with excess amounts (5 μg/mL) of EGFR antibodies. Effector cells in the peritoneum were identified using specific antibodies and their relative amount was related to constant amount of beads (Molecular Probed). For the depletion of PMNs, 250 μg of Gr-1 mAb (clone RB6.8C5, Ly6G/C-specific) or an isoype control (rat IgG1) was injected on Day −2 and Day 0. To deplete macrophages, 200 μL of chondronate liposomes (Free University, Amsterdam) were injected intraperitoneally on the day of the injection of tumour cells.

### A431-luc2 xenograft models

FcαRI Tg or WT littermate SCID mice were injected with 5 × 10^5^ A431-luc2 cells in the tail vein or in the peritoneum in 100 μL PBS. To increase the number of circulating PMNs all groups were injected with 20 μg PEG-G-CSF (Amgen) subcutaneously in the neck on Days −2 and Day 2 of the experiment. Tumour outgrowth was followed by serial BLI (PhotonImager, Biospace Lab). BLI images were processed using M3Vision software (Biospace Lab) and the images were edited in Adobe Photoshop. Cetuximab was injected once on Day 0, whereas IgA2 EGFR was injected daily between Day 0 and 4, in total five times. Circulating serum antibody levels were monitored by serial blood taking from alternating mice (two mice/time point).

### B16F10-luc2-EGFR model

B16F10 cells were lentivirally transduced with a luciferase-GFP construct and subsequently transfected with human EGFR. A clone was established that reproducibly grew in WT C57BL/6 mice. To increase the number of circulating PMNs all groups were injected subcutaneously with 20 μg PEG-G-CSF (Amgen) on Days −2 and Day 4 of the experiment. On day 0 1.5 × 10^5^ B16F10-luc2-EGFR cells were injected intravenously directly followed by intraperitoneal injection of 50 μg EGFR antibodies. Cetuximab was injected on Day 0 only, whereas 50 μg IgA2 EGFR was given 10 times daily to compensate for the shorted serum half-life. Tumour growth was monitored using serial BLI. At the end of the experiment mice were euthanized, lungs were removed and were scored for the number metastasis as described before (de Haij et al, 2010).

### Statistics

Data are represented as mean ± SEM and were analysed for significance using Student's unpaired *t*-test or Mann–Whitney test depending on the distribution of the data. Multiple groups were compared by ANOVA and further evaluated using Bonferroni's multiple comparison test using Graph Pad 5.0 software. A *p*-value <0.05 was considered statistically significant.

Despite clinical successes the efficacy of anti-cancer therapeutic mAbs needs further improvement. Currently, all antibodies are of the IgG isotype that engage Fcgamma receptors (FcγRs) to mediate activation of immune effector cells. *In vitro* results show that IgA anti-tumour antibodies are also able to mediate effective tumour cell killing. IgA antibodies engage Fcalpha receptor (FcαRI) that is expressed by different effector cells than FcγRs. However, the anti-tumour activity of IgA antibodies has not been tested *in vivo*.

RESULTS:

In this work, we report *in vivo* anti-cancer activity of IgA antibodies targeting epidermal growth factor receptor (EGFR) in both immunodeficient and immunocomptenent tumour models in mice. Since mice do not have FcαRI, we used mice transgenic for human FcαRI. We have found potent anti-tumour activity of IgA EGFR antibodies that greatly depended on the presence of human FcαRI transgene. We identified macrophages as the predominant effector cells mediating *in vivo* anti-tumour activity of IgA antibodies.

IMPACT:

These results demonstrate the anti-tumour activity of IgA antibodies *in vivo* and support the development of immunotherapeutic strategies against cancer based on targeting FcαRI.

## Author contributions

PB, SL, MN, JHMJ, GvT, LR and LPL performed research and analysed data. MD, MP, WKB, JGJW and LR designed research, analysed and interpreted data. LB and NvR contributed vital reagents. PB, SL, JHMJ, GvT, PWHIP, JGJW, TV and JHWL designed research and wrote the manuscript.

## Supporting Information

As a service to our authors and readers, this journal provides supporting information supplied by the authors. Such materials are peer reviewed and may be re-organized for online delivery, but are not copy-edited or typeset. Technical support issues arising from supporting information (other than missing files) should be addressed to the authors.

**Figure S1. ADCC assay with human PBMC.** (**A**) Specific killing of A1207 cells by human PBMC in a 4 h 51 Cr-release assay. (**B**) Specific killing of A1207 cells with 1 μg/mL EGFR antibodies by monocytes isolated from PBMC fractions and tested in a 4 h 51 Cr-release assay. Source data is available for this figure in the Supporting Information. **Figure S2. Cytofluorimetric analysis of G-CSF-primed mouse whole blood.** (**A**) Mice were injected subcutaneously with 20 μg of G-CSF and bled via the retro-orbital plexus four days later. Blood was analysed by cytofluorimetry and the number of effector cells was analysed relative to known amount of beads. (**B**) Expression of mouse FcγR and human FcαRI on different populations in the blood was analysed by staining with specific antibodies. Unstimulated and PEG-G-CSF-stimulated wild-type and FcαRI transgenic mice are compared. Source data is available for this figure in the Supporting Information. **Figure S3. *N*****-glycan sequencing profiles of antibodies *N*****-glycoprofiling of IgA1 EGFR (A) and IgA2 EGFR (B) antibodies: the reduced and alkylated antibodies were immobilized in a polyacrylamide gel block before releasing the *N*****-glycans by PNGaseF.** The glycans were fluorescently labelled with 2-AB and analysed on an ACQUITYUPLC-BEH-Glycan column. A combination of the retention times standardized to glucose units (GU) and exoglycosidase digestions were used to determine the sequence of the glycans. Source data is available for this figure in the Supporting Information. **Figure S4. Efficacy of depletion of specific cell types (A) PMN depletion efficacy.** WT Balb/c mice were injected with tumor cells and two times with 200 μg isotype control or with Gr-1 depleting antibody (Ly6G/C-specific). The effector cells from the peritoneal lavage were identified by FACS staining. (**B**) Macrophage depletion efficacy. WT Balb/c mice were injected with 200 μL chlodronate liposomes or PBS before the experiment to deplete macrophages. Mice received 50 μg IgA2 EGFR and the effector cells from the peritoneal lavage were identified by FACS staining. Source data is available for this figure in the Supporting Information. **Table S1.** Half-life of IgG1 and IgA1 EGFR. Source data is available for this figure in the Supporting Information. **Table S2.** A summary of the types of *N*-glycans on IgA1 EGFR and IgA2 EGFR. Source data is available for this figure in the Supporting Information.
