# Supplementary material for: IgA EGFR antibodies mediate tumour killing in vivo
Source: EMBO Mol Med. 2013 Aug 5;5(8):1213–26. doi: 10.1002/emmm.201201929 (PMC3944462; doi:10.1002/emmm.201201929)
Supplement: Supplementary file 1 — Figure S1. ADCC assay with human PBMC. (A) Specific killing of A1207 cells by human PBMC in a 4 h 51 Cr-release assay. (B) Specific killing of A1207 cells with 1 μg/mL EGFR antibodies by monocytes isolated from PBMC fractions and tested in a 4 h 51 Cr-release assay. Source data is available for this figure in the Supporting Information. Figure S2. Cytofluorimetric analysis of G-CSF-primed mouse whole blood. (A) Mice were injected subcutaneously with 20 μg of G-CSF and bled via the retro-orbital plexus four days later. Blood was analysed by cytofluorimetry and the number of effector cells was analysed relative to known amount of beads. (B) Expression of mouse FcγR and human FcαRI on different populations in the blood was analysed by staining with specific antibodies. Unstimulated and PEG-G-CSF-stimulated wild-type and FcαRI transgenic mice are compared. Source data is available for this figure in the Supporting Information. Figure S3. N-glycan sequencing profiles of antibodies N-glycoprofiling of IgA1 EGFR (A) and IgA2 EGFR (B) antibodies: the reduced and alkylated antibodies were immobilized in a polyacrylamide gel block before releasing the N-glycans by PNGaseF. The glycans were fluorescently labelled with 2-AB and analysed on an ACQUITYUPLC-BEH-Glycan column. A combination of the retention times standardized to glucose units (GU) and exoglycosidase digestions were used to determine the sequence of the glycans. Source data is available for this figure in the Supporting Information. Figure S4. Efficacy of depletion of specific cell types (A) PMN depletion efficacy. WT Balb/c mice were injected with tumor cells and two times with 200 μg isotype control or with Gr-1 depleting antibody (Ly6G/C-specific). The effector cells from the peritoneal lavage were identified by FACS staining. (B) Macrophage depletion efficacy. WT Balb/c mice were injected with 200 μL chlodronate liposomes or PBS before the experiment to deplete macrophages. Mice received 50 μg IgA2 EGFR and the [file emmm0005-1213-SD1.pdf]

SUPPORTING INFORMATION

Manuscript title: IgA EGFR antibodies mediate tumor killing in vivo

Peter Boross, Stefan Lohse, Maaike Nederend, J.H. Marco Jansen, Geert van Tetering, Michael Dechant, Matthias Peipp, Louise Royle, Li Phing Liew, Louis Boon, Nico van Rooijen, Wim Bleeker, Paul W.H.I. Parren, Jan G. van de Winkel, Thomas Valerius, Jeanette H.W. Leusen

Table of contents

Page 1: Supplementary Table 1 and 2
Page 2: Supplementary Fig 1 and 2
Page 3-4: Supplementary Fig 3
Page 4: Supplementary Fig 4

SUPPLEMENTARY TABLES

Supporting Information Table S1. Half-life of IgG1 and IgA1 EGFR

Antibody	*Estimated serum half-life in mice*
IgA1 EGFR	~15 hours
IgA2 EGFR	~15 hours
Cetuximab	> 4 days

Supporting Information Table S2. A summary of the types of *N*-glycans on IgA1 EGFR and IgA2 EGFR.

Type of structure	*IgA1 EGFR*	*IgA2 EGFR*
	Percentage of structures	
Sialylated	0.00	20.28
Core fucosylated	40.33	46.38
Man5	10.47	17.96
Mono-antennary	13.42	8.20
Bi-antennary	54.88	50.80
Tri-antennary	4.62	5.55
Tetra-antennary	4.58	3.62

SUPPLEMENTARY FIGURES

Figure S1

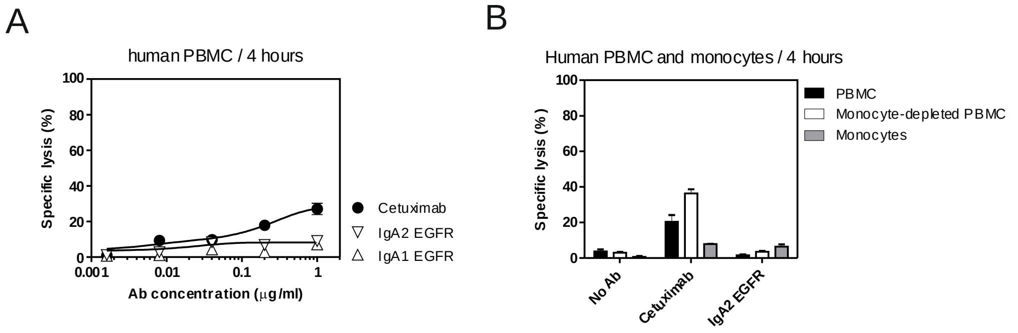

Supporting Information Fig S1. ADCC assay with human PBMC

A) Specific killing of A1207 cells by human PBMC in a 4 hours ^{51}Cr-release assay.

B) Specific killing of A1207 cells with 1 μg/ml EGFR antibodies by monocytes isolated from PBMC fractions and tested in a 4 hours ^{51}Cr-release assay.

Figure S2

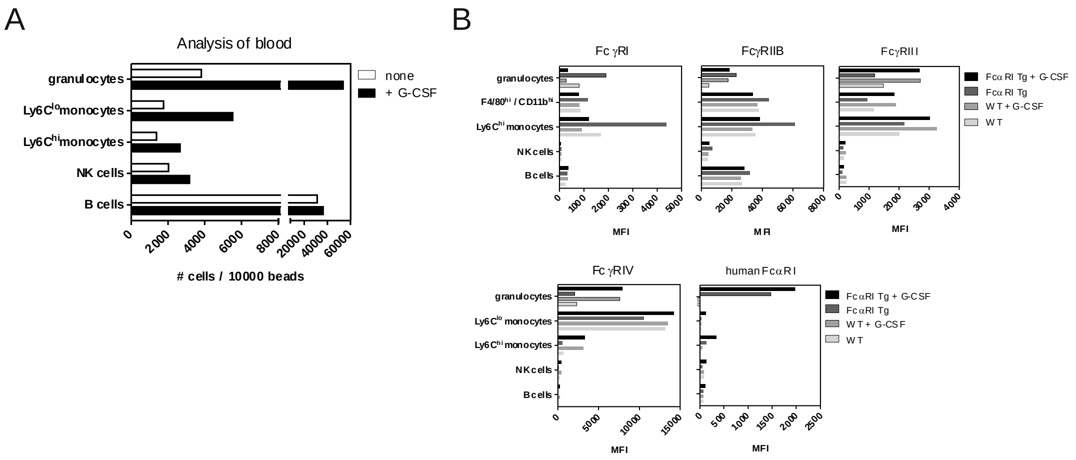

Supporting Information Fig S2. Cytofluorimetric analysis of G-CSF-primed mouse whole blood

A) Mice were injected subcutaneously with 20 μg of G-CSF and bled via the retro-orbital plexus four days later. Blood was analyzed by cytofluorimetry and the number of effector cells was analyzed relative to known amount of beads.

B) Expression of mouse FcγR and human FcαRI on different populations in the blood was analyzed by staining with specific antibodies. Unstimulated and PEG-G-CSF-stimulated wild type and FcαRI transgenic mice are compared.

Supporting Information Fig S3A.

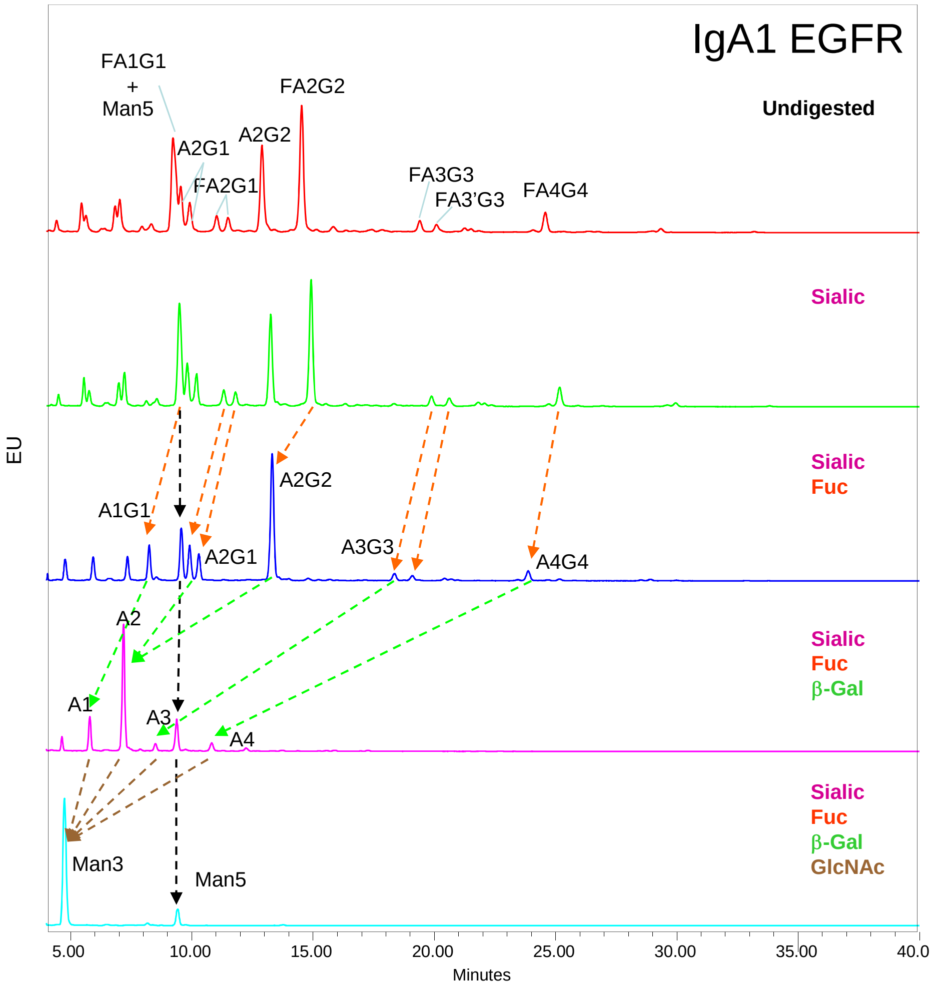

Supporting Information Fig S3B.

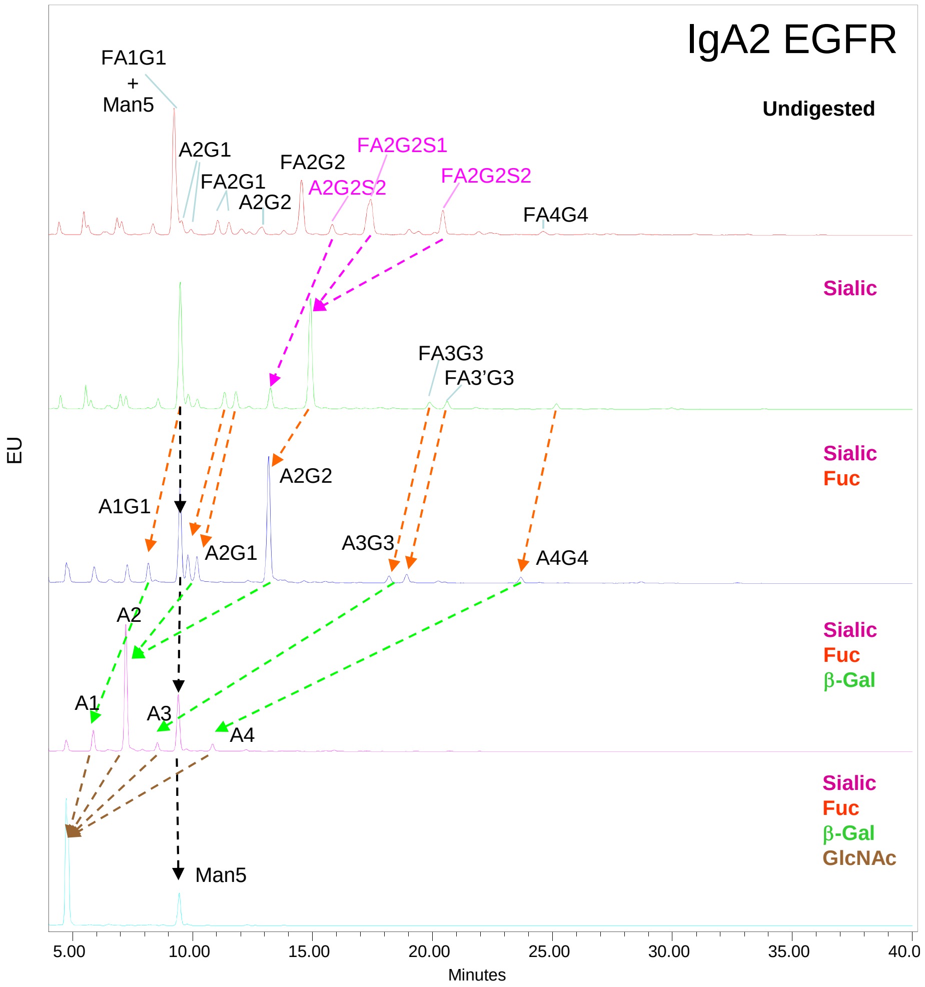

Supporting Information Fig S3. *N*-glycan sequencing profiles of antibodies *N*-glycoprofiling of IgA1 EGFR (A) and IgA2 EGFR (B) antibodies: The reduced and alkylated antibodies were immobilized in a polyacrylamide gel block before releasing the *N*-glycans by PNGaseF. The glycans were fluorescently labeled with 2-AB and analyzed on an ACQUITYUPLC-BEH-Glycan column. A combination of the retention times standardized to glucose units (GU) and exoglycosidase digestions were used to determine the sequence of the glycans.

The exoglycosidases used were:
Sialidase from *Arthrobacter ureafaciens* (Sialic) specific for α2-3, 6, 8, 9 sialic acids.
Beta Galactosidase from *Streptococcus pneumonia* (β-Gal) specific for β1-4 galactose.
Fucosidase from Bovine kidney (Fuc): specific for α1-6>2 fucose.
N-acetylglucosaminidase from *Streptococcus pneumoniae* (GlcNAc): specific for beta GlcNAc.

Structure abbreviations
All *N*-glycans have two core GlcNAcs; F at the start of the abbreviation indicates a core fucose α1-6 linked to the inner GlcNAc; Manx, number (x) of mannose on core GlcNAcs; Ax, number of antenna (GlcNAc) on trimannosyl core; A2, biantennary with both GlcNAcs as β1-2 linked; A3, triantennary with a GlcNAc linked β1-2 to both mannose and the third GlcNAc linked β1-4 to the α1-3 linked mannose; A4, GlcNAcs linked as A3 with additional GlcNAc β1-6 linked to α1-6 mannose; Gx, number (x) of galactose β1-4 linked to the antenna; Sx, number (x) of sialic acids linked to galactose.

Figure S4

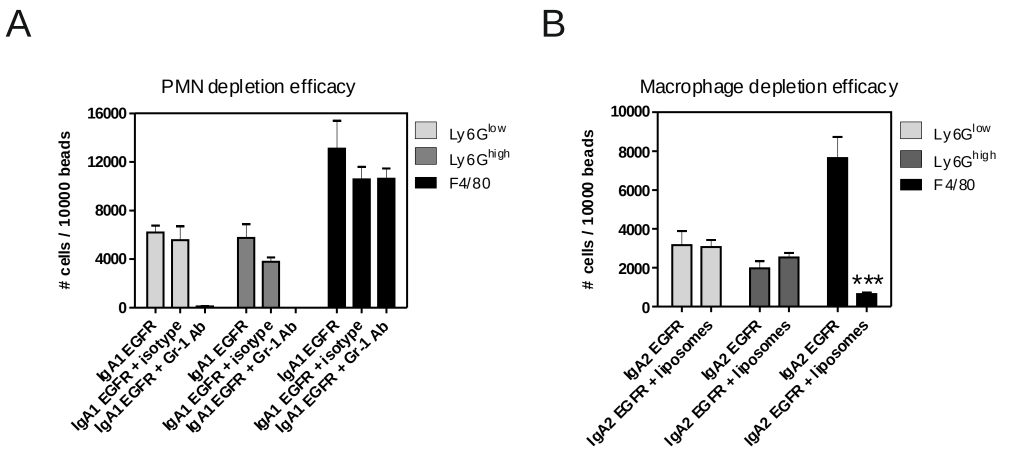

Supporting Information Fig S4. Efficacy of depletion of specific cell types
A) PMN depletion efficacy. WT Balb/c mice were injected with tumor cells and two times with 200 μg isotype control or with Gr-1 depleting antibody (Ly6G/C-specific). The effector cells from the peritoneal lavage were identified by FACS staining.
B) Macrophage depletion efficacy. WT Balb/c mice were injected with 200 μl chlodronate liposomes or PBS before the experiment to deplete macrophages. Mice received 50 μg IgA2 EGFR and the effector cells from the peritoneal lavage were identified by FACS staining.
